# Comparative Study on the Hardness, Adhesiveness, and Cohesiveness of Ingredients on the Basis of IDDSI Levels and Ingredient Selection

**DOI:** 10.1002/fsn3.71467

**Published:** 2026-01-19

**Authors:** Muxi Chen, Juan Duan, Yi Cheng, Jiuming Yan, Lei Shi, Liu Yuan

**Affiliations:** ^1^ Department of Clinical Nutrition, West China Hospital Sichuan University Chengdu China; ^2^ Clinical Medicine College, College of Traditional Chinese Medicine Mianyang China

**Keywords:** adhesiveness, cohesiveness, dietary management, dysphagia, elderly nutrition, food texture modification, hardness, IDDSI, texture profile analysis

## Abstract

This study aimed to quantitatively characterize hardness, adhesiveness, and cohesiveness of foods commonly consumed by elderly Chinese individuals across International Dysphagia Diet Standardization Initiative (IDDSI) Levels 0–7, to examine within‐level heterogeneity among food categories, and to provide practical guidance for texture modification and food substitution in dysphagia diets. Twenty‐six representative ingredients frequently consumed by older adults were selected and prepared to IDDSI Levels 0–7 using standardized cooking, blending, and dilution procedures. Texture Profile Analysis (TPA) was applied to measure hardness, adhesiveness, and cohesiveness, in conjunction with IDDSI syringe flow and fork pressure tests for level classification. As texture data were non‐normally distributed, differences across IDDSI levels and food categories were analyzed using the Kruskal–Wallis test with Dunn–Bonferroni post hoc correction (*α* = 0.05). Effect sizes were quantified using Cliff's *δ*, and within‐level interchangeability was evaluated on the basis of Euclidean centroid distance. All three texture parameters differed significantly across IDDSI levels (hardness *H* = 412.43; adhesiveness *H* = 238.89; cohesiveness *H* = 312.76; all *p* < 0.05). Hardness increased progressively from Level 0 to Level 7, cohesiveness declined accordingly, and adhesiveness exhibited a non‐linear pattern with a pronounced peak at intermediate levels (Levels 3–5). Within‐level analyses revealed significant category‐dependent heterogeneity (*p* < 0.05). Fiber‐rich vegetables and legumes showed higher adhesiveness and lower cohesiveness compared with protein‐based foods such as meats and eggs, suggesting a greater potential for oral residue. Interchangeability assessment indicated that foods at Levels 0–2 were largely functionally similar, whereas substantial divergence was observed at Levels 3–6, with centroid distances exceeding 0.60. Instrumental texture analysis demonstrated clear quantitative gradients across IDDSI levels and structured heterogeneity within the same level, confirming that foods sharing an IDDSI classification are not necessarily functionally equivalent. The proposed texture‐based substitution framework (centroid distance ≤ 0.60) offers an objective tool for optimizing dysphagia diet design in both clinical and home settings. These findings bridge the IDDSI system with naturally prepared Chinese foods and provide evidence to support culturally adaptable, texture‐based dietary management for elderly individuals with swallowing difficulties.

## Graphical abstract

This study quantified hardness, adhesiveness, and cohesiveness of 26 elderly‐preferred Chinese ingredients prepared to IDDSI Levels 0–7. We found clear cross‐level texture gradients but pronounced within‐level heterogeneity, showing that foods assigned to the same IDDSI level are not functionally interchangeable. A texture‐based substitution model using centroid distance (≤ 0.60) identifies safe versus high‐risk ingredient pairs, providing data‐driven guidance for culturally adapted dysphagia diets in clinical and home settings.

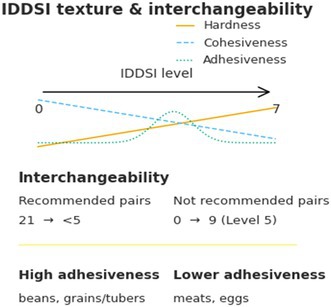

## 1 Introduction

Dysphagia, characterized by impaired swallowing safety and efficiency, is highly prevalent among older adults, stroke survivors, and patients with head and neck or neurodegenerative diseases (Liu et al. 2023; Ren et al. 2020; China Stroke Prevention Report 2020). In China, epidemiological surveys indicate that approximately 66.0% of elderly individuals experience swallowing difficulties, with prevalence rates of 21.0% in those aged 60–69, 28.0% in those aged 70–79, and 41.0% in individuals aged 80 years and above (Ren et al. 2020). The incidence of dysphagia following treatment for head and neck malignancies ranges from 50% to 60%, whereas post‐stroke dysphagia occurs in 37%–78% of cases (China Stroke Prevention Report 2020). Untreated dysphagia can result in malnutrition, dehydration, aspiration pneumonia, and impaired quality of life, leading to increased morbidity, prolonged hospitalization, and significant healthcare burdens (Chinese Expert Consensus Group on Swallowing Disorder Rehabilitation Assessment and Treatment 2019; The State Council Information Office of the People's Republic of China 2021). Accordingly, the development of safe, nutritious, and texture‐appropriate foods is central to nutritional management and rehabilitation for these patients.

To standardize terminology and clinical practice, the International Dysphagia Diet Standardization Initiative (IDDSI) developed a global framework defining eight levels (0–7) of texture modification on the basis of measurable flow and compression characteristics (Cichero et al. 2017; Dong et al. 2023). Although IDDSI has been widely adopted in hospitals and aged‐care institutions, most classification studies still rely on subjective sensory evaluations or commercial thickened formulations, and relatively few have systematically measured objective textural properties (e.g., hardness, adhesiveness, and cohesiveness) across the full range of IDDSI levels (Wong et al. 2023; Hadde et al. 2022; Xie et al. 2018). Recent quantitative studies have begun linking instrumental texture data with IDDSI classifications (Su et al. 2018; Giura et al. 2021; Godschalk‐Broers et al. 2022), yet substantial variability remains because of differences in formulation strategies, processing methods, and regional dietary practices.

Importantly, the IDDSI framework was originally designed to evaluate final food products intended for dysphagia patients, typically involving controlled texturization techniques—such as starch‐ or gum‐based thickeners, hydrocolloid gels, or high‐moisture restructured matrices (Fu et al. 2023; Tong et al. 2023; Wang et al. 2019). However, in real‐world home‐care settings, especially in China, most foods consumed by older adults are home‐prepared or minimally processed, lacking standardized structuring systems. The scientific validity of applying the IDDSI framework to such naturally cooked or hand‐prepared foods remains underexplored. Understanding whether everyday ingredients, when blended and prepared under controlled household conditions, can achieve measurable and reproducible texture differences across IDDSI levels is crucial for bridging daily cooking practices with standardized dysphagia management (Muxi et al. 2024; Chen, Cheng, et al. 2025; Chen, Mu, et al. 2025).

Food texture is influenced by multiple intrinsic and extrinsic factors—moisture content, blending duration, temperature, and macronutrient composition (particularly protein and fiber) (Qin et al. 2025; Hernández et al. 2025; Lu et al. 2025). Systematic comparison of diverse food categories under controlled preparation conditions can reveal how ordinary ingredients approximate IDDSI‐defined texture boundaries, and whether specific ingredient types inherently align with safer swallowing properties. Nevertheless, few studies have simultaneously evaluated cross‐level (0–7) and within‐level variations among different food categories, particularly using foods preferred by elderly Chinese individuals, whose dietary culture emphasizes fresh, lightly processed, and naturally textured foods.

Therefore, the present study aimed to quantitatively compare the hardness, adhesiveness, and cohesiveness of 26 commonly consumed ingredients across IDDSI levels 0–7, focusing on both cross‐level differences and within‐level variations among food categories (meats, vegetables, fruits, grains/tubers, eggs, and mixed beans). Rather than replacing standardized formulation‐based studies, this work sought to provide foundational data for understanding how naturally prepared foods behave under the IDDSI classification, thereby informing practical, culturally relevant dietary strategies for dysphagia care. This approach complements previous formulation research and supports the future development of scientifically validated, dysphagia‐friendly diets grounded in both instrumental evidence and regional dietary habits.

## 2 Methods

### 2.1 Food Data Screening

To comprehensively characterize food preferences among elderly individuals in China, a large‐scale web‐based screening was conducted using Python (v3.11) in combination with the Scrapy web‐crawling framework. Recipe data were extracted from the Chinese cuisine platform Meishichina (www.meishichina.com), one of the most widely used online cooking databases in China. The screening covered seven major geographical regions (Northeast, North, East, South, Central, Southwest, and Northwest China) and focused specifically on recipes labeled or recommended for elderly populations.

All data collection procedures complied with the website's access policies and relevant national data management regulations. Extracted information included recipe names, ingredient lists, and basic preparation methods. Following the collection, the dataset was systematically cleaned to remove duplicate entries, advertisements, and irrelevant content. Only core ingredient information was retained for subsequent analysis. This data‐driven screening process provided a robust and culturally representative foundation for identifying commonly consumed food ingredients among elderly Chinese individuals.

### 2.2 Selection of Experimental Materials

On the basis of the web‐derived recipe database and nutritional recommendations for elderly populations, a total of 26 food ingredients with the highest occurrence frequency in elderly‐oriented Chinese recipes were selected as experimental materials. These ingredients were chosen to comprehensively represent the major food categories commonly consumed by older adults in China, ensuring both cultural relevance and dietary representativeness.

Specifically, the selected ingredients covered six primary categories: meats (pork, beef, chicken, and fish), eggs (hen's eggs), grains and tubers (rice, noodles, oats, potatoes, sweet potatoes, yams, and pumpkin), vegetables (tomato, eggplant, cabbage, carrot, Chinese greens, broccoli, and spinach), mixed beans (red beans and mung beans), fruits (apple, pear, peach, and banana), as well as tofu as a commonly consumed plant‐based protein source. The full list of ingredients, corresponding food categories, and selection frequency derived from the web‐based screening is provided in Table S1.

All ingredients were purchased fresh on the same day from local markets. Inedible portions were removed prior to processing, and all samples were prepared under standardized laboratory conditions to minimize texture variability associated with raw material handling. For each ingredient, three independent batches were prepared to ensure reproducibility and statistical robustness.

### 2.3 Food Processing Methods

Food samples were prepared using standardized domestic cooking procedures designed to simulate typical household preparation while maintaining experimental control. Briefly, all raw ingredients were washed and cut into uniform cubes (approximately 2 × 2 cm). Depending on ingredient type, samples were either steamed or boiled until fully softened. Cooked samples were subsequently homogenized using a laboratory food processor (Bear QSJ‐B03A1, China) to obtain smooth purees. Water was added at predefined weight ratios to generate a series of texture consistencies spanning multiple IDDSI levels.

To enhance clarity while avoiding procedural fragmentation, food‐specific preparation strategies were grouped by category. In general, meats were trimmed of visible sinew and fat, cooked thoroughly, and homogenized with incremental water addition to obtain purees of increasing dilution. Eggs were processed either as hard‐boiled yolk purees or as steamed egg custards, depending on target texture. Grains and tubers were pre‐soaked or fully cooked prior to homogenization to ensure starch gelatinization, whereas vegetables and mixed beans were softened by steaming following appropriate soaking when necessary. Fruits were processed either raw or lightly steamed before blending, depending on fiber structure. Representative ingredient‐to‐water ratios ranged from 1:0 to 1:2 (w/w), with exact ratios determined on the basis of ingredient structure and target IDDSI level.

Detailed, ingredient‐specific preparation parameters—including soaking duration, cooking method, cooking time, and dilution ratios—are systematically summarized in Table S2, enabling full reproducibility without disrupting narrative flow in the main text.

Across all preparations, the total mass of each processed sample was standardized to 50 g. Blending parameters were kept constant (blending time: 30 s; speed: 10,000 rpm), and all procedures were conducted under controlled environmental conditions (25°C ± 1°C; 50% ± 5% relative humidity). For each ingredient and target consistency, three replicate samples were prepared.

### 2.4 IDDSI Evaluation Methods

All food samples were classified according to the International Dysphagia Diet Standardization Initiative (IDDSI, 2019) framework, covering texture Levels 0–7. Standard IDDSI assessment tools, including a 10 mL syringe, stainless‐steel fork, and spoon, were used. All evaluations were conducted in a quiet environment under consistent illumination and room temperature (25°C).

For liquid and transitional textures (Levels 0–4), the IDDSI Flow Test was applied. A 10 mL syringe was filled with the test sample, the nozzle was occluded for 10 s, and the remaining volume was recorded to determine the corresponding IDDSI level. For solid and soft textures (Levels 5–7), fork, spoon, and finger pressure tests were performed to assess flowability, structural integrity, breakability, and cohesiveness in accordance with IDDSI guidelines.

Each sample was independently evaluated three times by two trained assessors. Final IDDSI levels were assigned by consensus, with borderline cases resolved following IDDSI decision rules. Inter‐rater reliability was high (Cohen's *κ* = 0.93), confirming excellent reproducibility of the classification process.

### 2.5 Texture Measurement Methods

Texture properties, including hardness, cohesiveness, and adhesiveness, were quantified using a texture analyzer (TA.XT Plus, Stable Micro Systems, UK) equipped with a 36 mm cylindrical probe (P/36R) and a 50 kg load cell. Texture Profile Analysis (TPA) was performed under standardized conditions, with double compression applied at a test speed of 0.50 mm/s to a target distance of 3.0 mm and a trigger force of 3.0 gf. All measurements were conducted at 25°C ± 1°C.

Each sample was tested in triplicate, and the probe was cleaned with 70% ethanol and air‐dried between measurements to prevent cross‐contamination. Hardness was defined as the maximum force during the first compression, adhesiveness as the negative area under the force–time curve during probe withdrawal, and cohesiveness as the ratio of the area under the second compression to that under the first compression. Data acquisition and processing were performed using Exponent Connect software (Stable Micro Systems).

Because texture data were not normally distributed, results are presented as medians with interquartile ranges (IQRs).

### 2.6 Statistical Analysis

Statistical analyses were conducted using SPSS Statistics 25.0 (IBM, USA) and Python (pandas, scipy, and scikit‐posthocs libraries). Descriptive statistics were used to summarize the distribution of food categories across IDDSI levels, with texture parameters expressed as median [IQR].

Differences in texture properties across IDDSI Levels 0–7 were evaluated using the Kruskal–Wallis test, followed by Dunn–Bonferroni post hoc comparisons when significant. Monotonic trends across IDDSI levels were assessed using the Jonckheere–Terpstra test. Within each IDDSI level, variability among food categories was examined using the Kruskal–Wallis test. Effect sizes were quantified using Cliff's delta (*δ*) and epsilon‐squared (*ε* ^2^) to identify food categories exhibiting significant deviations from the median texture profile.

Multiple linear regression analysis was performed to explore associations between IDDSI level and texture parameters (hardness, cohesiveness, and adhesiveness). Outliers (|*Z*| > 3) were excluded prior to model fitting. Bootstrap resampling (1000 iterations) was applied to estimate 95% confidence intervals for medians and effect sizes. Data visualization, including boxplots, radar charts, and isotonic regression curves, was generated using Matplotlib and Seaborn. All statistical tests were two‐tailed, with *p* ≤ 0.05 considered statistically significant.

## 3 Results

### 3.1 Descriptive Overview of Texture Parameters Across IDDSI Levels and Food Categories

**FIGURE 1:**
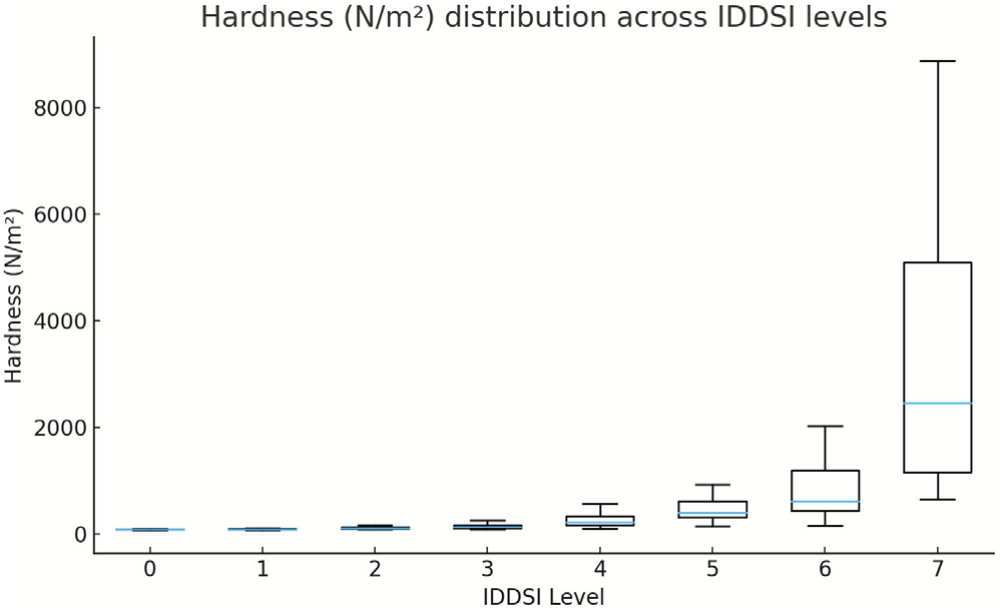
Hardness (N/m^2^) distribution across IDDSI levels (boxplots).

**FIGURE 2:**
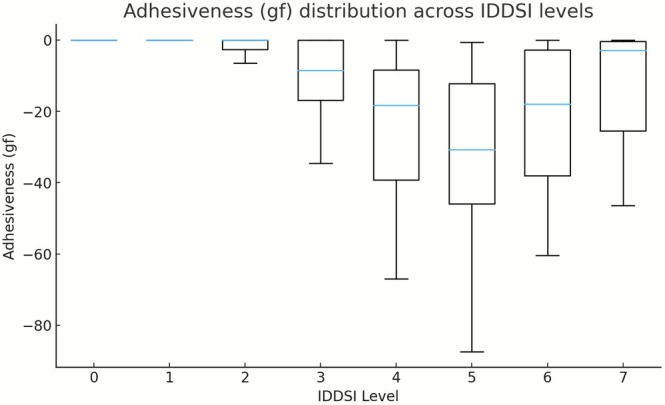
Adhesiveness (gf) distribution across IDDSI levels (boxplots).

**FIGURE 3:**
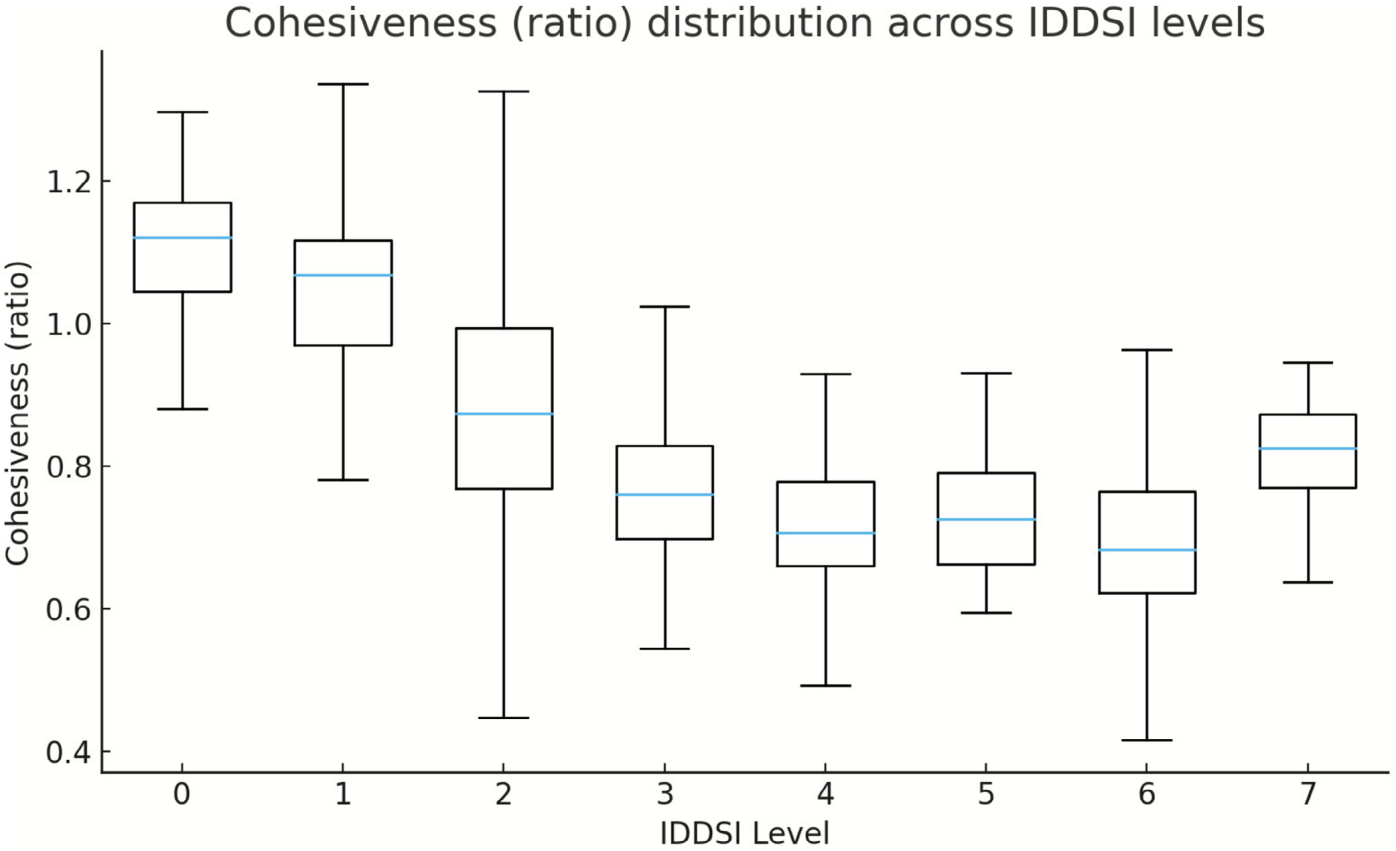
Cohesiveness (ratio) distribution across IDDSI levels (boxplots).

Across all samples, hardness, adhesiveness, and cohesiveness exhibited clear and systematic variations across IDDSI Levels 0–7, reflecting the progressive modification of food texture required to accommodate different swallowing abilities. Descriptive statistics for each texture parameter stratified by IDDSI level and food category are summarized in Table 1, and their distribution patterns are illustrated in Figures 1, 2, 3.

**TABLE 1:** Descriptive statistics of hardness, adhesiveness, and cohesiveness across IDDSI levels and food categories (Median [IQR], Min–Max).

IDDSI	Category	*n*	Hardness (Median [IQR])	Hardness (Min–Max)	Adhesiveness (Median [IQR])	Adhesiveness (Min–Max)	Cohesiveness (Median [IQR])	Cohesiveness (Min–Max)
0	Other (tofu)	12	82.18 [81.26 to 84.10]	80.24 to 86.88	0.00 [0.00 to 0.00]	0.00 to 0.00	1.09 [1.04 to 1.12]	1.00 to 1.17
0	Mixed beans	3	87.60 [85.15 to 88.59]	82.70 to 89.58	0.00 [0.00 to 0.00]	0.00 to 0.00	1.16 [1.13 to 1.17]	1.10 to 1.18
0	Fruits	36	84.91 [81.83 to 91.58]	78.88 to 138.34	0.00 [0.00 to 0.00]	−0.16 to 0.00	1.11 [1.02 to 1.17]	0.80 to 1.30
0	Meats	15	84.08 [81.42 to 85.32]	79.27 to 87.26	0.00 [0.00 to 0.00]	0.00 to 0.00	1.14 [1.02 to 1.18]	0.96 to 1.19
0	Vegetables	29	86.20 [82.93 to 89.02]	77.72 to 100.72	0.00 [0.00 to 0.00]	0.00 to 0.00	1.13 [1.08 to 1.16]	0.92 to 1.45
0	Eggs	6	84.44 [81.85 to 88.01]	80.64 to 118.36	0.00 [0.00 to 0.00]	0.00 to 0.00	1.12 [1.09 to 1.17]	0.99 to 1.19
0	Grains and tubers	9	84.93 [83.85 to 85.69]	82.17 to 86.38	0.00 [0.00 to 0.00]	0.00 to 0.00	1.13 [1.10 to 1.20]	1.06 to 1.20
1	Other (tofu)	3	91.74 [91.50 to 91.82]	91.27 to 91.90	0.00 [0.00 to 0.00]	0.000 to 0.000	0.992 [0.97 to 1.01]	0.94 to 1.04
1	Mixed beans	3	86.25 [85.73 to 87.39]	85.21 to 88.54	0.00 [0.00 to 0.00]	0.000 to 0.000	1.13 [1.11 to 1.13]	1.10 to 1.14
1	Fruits	24	88.36 [84.48 to 115.72]	81.95 to 179.77	0.00 [−0.81 to 0.00]	−14.76 to 0.00	1.05 [0.77 to 1.10]	0.66 to 1.34
1	Vegetables	33	93.12 [88.89 to 96.96]	78.54 to 109.28	0.00 [0.00 to 0.00]	−0.52 to 0.00	1.05 [0.94 to 1.11]	0.00 to 1.27
1	Grains and tubers	21	87.25 [83.47 to 87.87]	81.71to 93.56	0.00 [0.00 to 0.00]	0.00 to 0.00	1.12 [1.09 to 1.18]	0.99 to 1.26
2	Other (tofu)	3	103.83 [103.67 to 104.34]	103.50 to 104.85	−2.13 [−2.1 to −1.42]	−2.18 to −0.70	0.72 [0.72 to 0.73]	0.71 to 0.74
2	Mixed beans	6	94.37 [91.26 to 96.73]	87.47 to 98.28	0.00 [0.00 to 0.00]	0.00 to 0.00	1.05 [0.98 to 1.13]	0.90 to 1.15
2	Fruits	15	118.36 [92.56 to 258.27]	89.96 to 372.68	−0.14 [−30.64 to 0.00]	−40.02 to 0.00	0.87 [0.78 to 0.94]	0.72 to 1.58
2	Meats	6	86.14 [83.86 to 88.72]	80.34 to 89.60	0.00 [0.00 to 0.00]	0.00 to 0.00	1.10 [1.07 to 1.11]	1.05 to 1.13
2	Vegetables	21	104.88 [100.44 to 112.78]	89.21 to 135.13	0.00 [−0.79 to 0.00]	−7.29 to 0.00	0.90 [0.79 to 0.94]	0.73 to 1.00
2	Eggs	15	253.16 [153.16 to 447.32]	105.65 to 593.64	−6.43 [−11.1 to −1.97]	−16.74 to −0.01	0.72 [0.52 to 0.82]	0.45 to 0.87
2	Grains and tubers	12	98.92 [89.87 to 101.18]	81.79 to 106.64	0.00 [−0.12 to 0.00]	−1.06 to 0.00	1.01 [0.86 to 1.10]	0.76 to 1.26
3	Other (tofu)	6	214.48 [176.57 to 249.73]	169.37 to 258.13	−20.48 [−22.11 to −16.95]	−24.99 to −15.56	0.61 [0.60 to 0.63]	0.58 to 0.66
3	Mixed beans	6	161.60 [116.89 to 207.31]	115.81 to 211.98	−7.48 [−15.70 to −0.23]	−19.09 to −0.03	0.67 [0.57 to 0.77]	0.54 to 0.81
3	Fruits	12	156.03 [132.85 to 225.52]	116.61 to 315.66	−27.35 [−32.95 to −18.85]	−55.73 to −8.47	0.75 [0.74 to 0.76]	0.71 to 0.83
3	Meats	6	109.75 [103.41 to 114.70]	100.05 to 116.36	0.00 [−0.08 to 0.00]	−0.25 to 0.00	0.88 [0.81 to 0.96]	0.79 to 0.98
3	Vegetables	24	138.11 [118.38 to 162.96]	98.20 to 207.38	−6.47 [−14.15 to −1.03]	−29.85 to 0.00	0.74 [0.70 to 0.79]	0.59 to 1.36
3	Eggs	3	179.39 [159.03 to 211.92]	138.67 to 244.46	−15.59 [−17.00 to −15.07]	−18.41 to −14.55	0.71 [0.68 to 0.71]	0.66 to 0.71
3	Grains and tubers	24	108.00 [96.10 to 127.73]	85.83 to 165.35	−1.33 [−8.95 to 0.00]	−16.92 to 0.00	0.87 [0.77 to 1.00]	0.65 to 1.16
4	Mixed beans	3	299.10 [285.98 to 302.44]	272.86 to 305.79	−26.94 [−27.45 to −24.73]	−27.96 to −22.51	0.51 [0.50 to 0.51]	0.49 to 0.51
4	Fruits	3	98.36 [94.99 to 100.05]	91.63 to 101.73	−1.77 [−1.94 to −0.91]	−2.10 to −0.04	0.79 [0.79 to 0.86]	0.78 to 0.93
4	Meats	3	124.07 [120.32 to 125.33]	116.56 to 126.60	0.00 [0.00 to 0.00]	0.00 to 0.00	0.90 [0.89 to 0.90]	0.89to 0.90
4	Vegetables	21	183.17 [169.38 to 297.13]	150.79 to 379.09	−19.02 [−53.13 to −10.13]	−67.00 to −6.16	0.70 [0.68 to 0.79]	0.61 to 0.88
4	Eggs	12	367.96 [254.92 to 605.40]	215.96 to 1624.02	−12.65 [−17.08 to −5.88]	−55.97 to −0.15	0.69 [0.56 to 0.75]	0.47 to 0.76
4	Grains and tubers	13	212.78 [176.16 to 340.47]	152.55 to 684.73	−31.37 [−45.39 to −19.66]	−59.14 to −15.04	0.69 [0.66 to 0.78]	0.62 to 0.81
5	Other (tofu)	6	374.12 [311.01 to 414.27]	267.61 to 424.46	−12.23 [−14.73 to −9.46]	−15.47 to −3.99	0.65 [0.64 to 0.68]	0.63 to 0.75
5	Mixed beans	6	725.13 [440.79 to 1038.02]	275.57 to 1100.91	−104.49 [−132.53 to −53.78]	−152.18 to −45.89	0.66 [0.64 to 0.71]	0.63 to 0.74
5	Fruits	3	146.12 [145.74 to 147.71]	145.36 to 149.31	−1.40 [−1.64 to −0.99]	−1.87 to −0.58	1.11 [1.10 to 1.40]	1.10 to 1.70
5	Meats	12	497.28 [348.49 to 631.78]	226.80 to 669.27	−6.50 [−10.99 to −2.46]	−21.88 to −1.85	0.70 [0.64 to 0.77]	0.62 to 0.86
5	Vegetables	12	369.25 [322.01 to 413.23]	312.05 to 603.90	−34.31 [−38.20 to −30.92]	−46.68 to 27.09	0.74 [0.72 to 0.77]	0.70 to 0.80
5	Eggs	3	1692.79 [1580.90 to 1935.43]	1469.10 to 2178.07	−30.63 [−31.82 to −24.57]	−33.00 to −18.50	0.61 [0.60 to 0.64]	0.59 to 0.68
5	Grains and tubers	21	400.35 [328.04 to 729.72]	193.36 to 1169.54	−45.88 [−63.86 to −27.84]	−87.38 to −9.12	0.76 [0.69 to 0.86]	0.63 to 1.48
6	Other (tofu)	6	1859.94 [1608.38 to 1999.26]	1085.01 to 2643.17	−31.250 [−37.572 to −23.165]	−49.89 to −3.37	0.86 [0.86 to 0.86]	0.86 to 0.88
6	Mixed beans	12	637.70 [586.23 to 897.28]	466.30 to 1377.17	−1.905 [−9.992 to −0.323]	−35.29 to −0.07	0.60 [0.53 to 0.64]	0.43 to 0.68
6	Fruits	9	588.95 [349.08 to 1478.00]	309.22 to 1776.23	−2.80 [−3.17 to −1.95]	−15.99 to −1.23	0.71 [0.68 to 0.83]	0.66 to 0.86
6	Meats	3	610.91 [586.82 to 617.18]	562.72 to 623.44	−20.76 [−22.94 to −17.40]	−25.11 to −14.04	0.69 [0.68 to 0.69]	0.66 to 0.70
6	Vegetables	21	656.94 [382.63 to 917.64]	158.05 to 1650.82	−11.07 [−32.99 to −3.58]	−52.07 to −1.37	0.66 [0.54 to 0.72]	0.40 to 0.77
6	Eggs	6	2573.20 [538.85 to 4617.10]	352.00 to 8722.39	−9.13 [−23.68 to −0.32]	−33.78 to −0.02	0.67 [0.65 to 0.69]	0.60 to 0.78
6	Grains and tubers	21	479.06 [311.13 to 723.85]	211.09 to 1447.21	−43.77 [−60.39 to −34.09]	−149.53 to −2.52	0.75 [0.67 to 0.91]	0.00 to 0.96
7	Mixed beans	3	1242.23 [1218.6 to 1471.55]	1195.12 to 1700.87	−0.54 [−0.58 to −0.35]	−0.62 to 0.15	0.51 [0.50 to 0.53]	0.48 to 0.56
7	Fruits	12	6699.58 [5299.7 to 9671.19]	3621.03 to 14822.36	−20.44 [−36.53 to −2.25]	−46.37 to −0.29	0.88 [0.83 to 0.89]	0.75 to 0.95
7	Meats	3	1050.77 [1013.9 to 1055.59]	977.22 to 1060.42	−0.22 [−0.28 to −0.11]	−0.33 to 0.01	0.77 [0.77 to 0.77]	0.76 to 0.77
7	Vegetables	6	1649.70 [772.54 to 2816.05]	655.20 to 3320.15	−5.99 [−21.35 to −0.43]	−36.72 to −0.26	0.70 [0.56 to 0.79]	0.49 to 0.84
7	Eggs	3	744.05 [718.39 to 788.63]	692.73 to 833.22	−0.42 [−0.83 to −0.42]	−1.24 to −0.41	0.91 [0.90 to 0.93]	0.88 to 0.94
7	Grains and tubers	9	1927.13 [1665.6 to 2723.51]	1285.05 to 2946.28	−16.06 [−25.42 to −10.88]	−28.94 to −0.30	0.84 [0.80 to 0.86]	0.77 to 0.87

Hardness demonstrated a pronounced and monotonic increase with advancing IDDSI level (Figure 1). Median hardness values were consistently low at Levels 0–1, corresponding to liquid and thin semi‐liquid textures, and increased substantially from Level 3 onward, reaching the highest values at Levels 6–7. This progressive escalation indicates that hardness is closely aligned with the IDDSI framework and represents a primary mechanical discriminator between texture levels. Despite this overall trend, substantial variability was observed among food categories within the same IDDSI level. At Level 0, hardness values were uniformly low across all categories, with narrow interquartile ranges. In contrast, intermediate and advanced levels exhibited marked heterogeneity. For example, at Level 5, eggs and mixed beans showed markedly higher median hardness and wider interquartile ranges than fruits and vegetables. At Level 7, fruits exhibited the highest median hardness values, whereas meats and eggs maintained comparatively lower hardness within the same level (Table 1), highlighting pronounced within‐level heterogeneity.

Adhesiveness displayed a distinct non‐linear pattern across IDDSI levels (Figure 2). At Levels 0–1, adhesiveness values were consistently close to zero across all food categories, reflecting minimal resistance during probe withdrawal in liquid and thin semi‐liquid samples. From Level 2 to Level 5, adhesiveness became progressively more negative, indicating increased surface stickiness associated with transitional textures. The most negative median adhesiveness values were observed at Level 5, particularly in mixed beans and grains/tubers. At higher IDDSI levels (Levels 6–7), absolute adhesiveness values decreased again, suggesting reduced surface stickiness as food structure transitioned toward more cohesive and solid‐like forms. Variability in adhesiveness also increased from Level 3 onward, with wider interquartile ranges observed across multiple food categories (Table 1).

Cohesiveness generally decreased with increasing IDDSI level, with the highest values observed at Levels 0–1 and lower values predominating at Levels 5–6 (Figure 3). Liquids and semi‐liquids exhibited cohesiveness values close to or exceeding unity, reflecting continuous internal structure under compression. In contrast, most samples at Levels 5–6 showed reduced cohesiveness, consistent with the formation of particulate or transitional textures. At Level 7, cohesiveness values increased slightly and displayed greater between‐category variability. Notably, mixed beans exhibited the lowest median cohesiveness at this level, whereas eggs maintained comparatively high cohesiveness (Table 1), indicating differences in internal structural integrity among solid foods that met the same IDDSI classification.

Taken together, these descriptive results demonstrate that texture parameters exhibit both systematic level‐dependent trends and pronounced food‐category‐specific variability. Although the IDDSI level effectively captures the overall progression of texture modification, substantial heterogeneity persists within individual levels, underscoring the necessity of food‐specific texture characterization beyond categorical classification.

### 3.2 Cross‐Level Differences in Hardness, Adhesiveness, and Cohesiveness Across IDDSI Levels

To quantitatively evaluate texture differences across IDDSI Levels 0–7, non‐parametric statistical analyses were performed for hardness, adhesiveness, and cohesiveness. Overall cross‐level comparisons using the Kruskal–Wallis test revealed highly significant differences for all three texture parameters (*p* < 0.05), accompanied by large effect sizes (*η* ^2^ = 0.81 for hardness, 0.54 for adhesiveness, and 0.67 for cohesiveness), indicating that IDDSI level accounted for a substantial proportion of variability in texture properties (Table 2). Monotonic trends across IDDSI levels were further examined using the Jonckheere–Terpstra test. Significant ordered trends were confirmed for all parameters, with hardness increasing progressively across levels, whereas adhesiveness and cohesiveness exhibited significant decreasing trends (Table 2). These findings support the existence of systematic, level‐dependent texture modification consistent with the IDDSI framework.

**TABLE 2:** Cross‐level comparisons of texture parameters among IDDSI levels (Kruskal–Wallis, Jonckheere–Terpstra).

Parameter	Kruskal–Wallis *H*	*p* (KW)	*η* ^2^ (KW)	Jonckheere–Terpstra *Z*	*p* (JT)
Hardness (N/m^2^)	412.43	< 0.05	0.81	−19.74	< 0.05
Adhesiveness (gf)	238.89	< 0.05	0.54	−10.84	< 0.05
Cohesiveness (ratio)	312.76	< 0.05	0.67	−15.66	< 0.05

To identify where texture transitions occurred along the IDDSI continuum, pairwise comparisons between adjacent levels were conducted using the Dunn–Bonferroni procedure (Table 3). Hardness differed significantly between all adjacent IDDSI levels (*p* < 0.05), with small‐to‐moderate effect sizes (Cliff's *δ* ranging from −0.17 to −0.27), indicating a consistent stepwise increase in resistance to compression as IDDSI level increased. In contrast, adhesiveness showed a more selective pattern of change. No significant difference was observed between Levels 0 and 1 (*p* > 0.05), whereas significant differences emerged from Level 1–2 onward. The largest change in adhesiveness occurred between Levels 1 and 2 (*Z* = −5.98, *δ* = −0.48), suggesting a pronounced transition in surface stickiness as foods moved from thin liquids to slightly thicker textures. Subsequent adjacent‐level comparisons remained statistically significant but were associated with smaller effect sizes, indicating more gradual changes at higher levels. Cohesiveness also demonstrated significant differences between most adjacent IDDSI levels, with the exception of Levels 0–1 (*p* > 0.05). The strongest contrast was observed between Levels 1 and 2 (*δ* = 0.51), followed by progressively smaller effect sizes across higher levels. This pattern indicates that major changes in internal structural integrity occur early in the IDDSI progression, whereas later transitions are characterized by more incremental modifications.

**TABLE 3:** Dunn–Bonferroni pairwise comparisons between adjacent IDDSI levels for texture parameters.

Parameter	Level (i–j)	*Z*	*p* (Bonf.)	Cliff's *δ*
Hardness (N/m^2^)	0–1	−2.13	< 0.05	−0.17
	1–2	−3.79	< 0.05	−0.23
	2–3	−3.88	< 0.05	−0.26
	3–4	−3.27	< 0.05	−0.22
	4–5	−3.06	< 0.05	−0.21
	5–6	−3.95	< 0.05	−0.25
	6–7	−3.86	< 0.05	−0.27
Adhesiveness (gf)	0–1	−1.53	> 0.05	−0.11
	1–2	−5.98	< 0.05	−0.48
	2–3	−3.45	< 0.05	−0.28
	3–4	−2.11	< 0.05	−0.15
	4–5	−2.42	< 0.05	−0.18
	5–6	−1.94	< 0.05	−0.13
	6–7	−1.75	< 0.05	−0.12
Cohesiveness (ratio)	0–1	0.92	> 0.05	0.07
	1–2	6.45	< 0.05	0.51
	2–3	3.69	< 0.05	0.23
	3–4	3.31	< 0.05	0.21
	4–5	3.04	< 0.05	0.19
	5–6	2.89	< 0.05	0.18
	6–7	2.68	< 0.05	0.16

**FIGURE 4:**
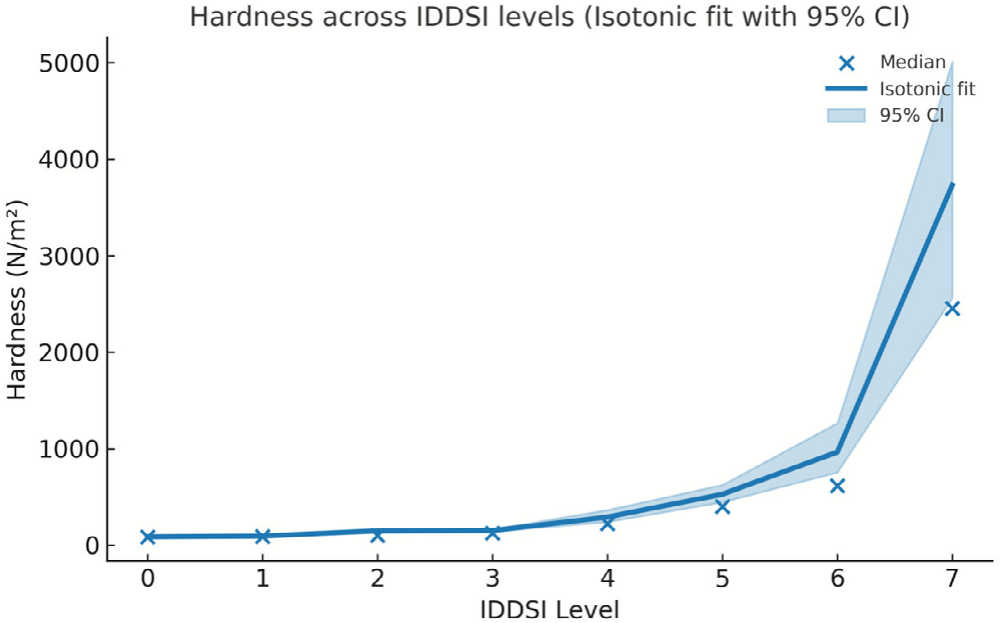
Hardness across IDDSI levels. (Isotonic regression fit with the 95% confidence interval).

**FIGURE 5:**
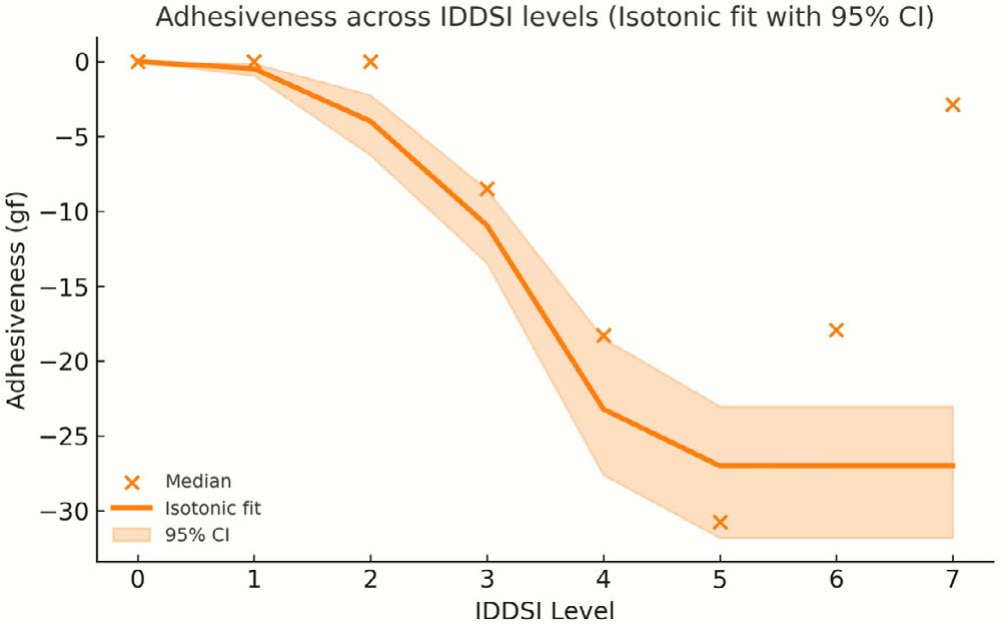
Adhesiveness across IDDSI levels. (Isotonic regression fit with the 95% confidence interval).

**FIGURE 6:**
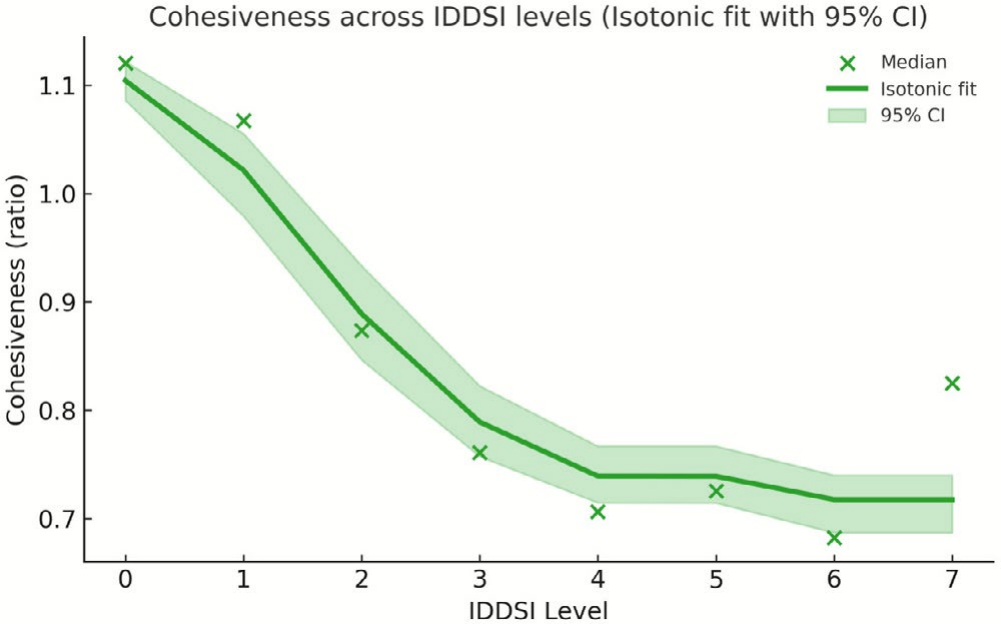
Cohesiveness across IDDSI levels. (Isotonic regression fit with the 95% confidence interval).

The fitted isotonic regression curves with 95% bootstrap confidence intervals further illustrate these level‐dependent trends (Figures 4, 5, 6). Hardness increased continuously with the advancing IDDSI level, whereas adhesiveness exhibited a marked decline after Level 2, and cohesiveness showed a gradual overall decrease from Level 0 to Level 7. Together, these visualizations reinforce the statistical findings and highlight the distinct transition points along the IDDSI scale.

### 3.3 Intra‐Level Variability Across Food Categories Within the Same IDDSI Level

To determine whether foods classified within the same IDDSI level exhibited interchangeable texture characteristics, intra‐level variability across food categories was systematically evaluated using non‐parametric statistical analyses. Kruskal–Wallis tests demonstrated that the three texture parameters—hardness, adhesiveness, and cohesiveness—showed markedly different degrees of variability across food categories depending on IDDSI level (Table 4).

**TABLE 4:** Within‐level Kruskal–Wallis tests for hardness, adhesiveness, and cohesiveness by IDDSI level (KW H, *p*, *ε* ^2^).

Level	Metric	KW *H*	KW *p*	Epsilon^2^	Categories(*n* ≥ 3)
0	Adhesiveness (gf)	8.45	> 0.05	0.024	Eggs, Fruits, Grains/Tubers, Meats, Mixed beans, Other, Vegetables
	Cohesiveness (ratio)	4.52	> 0.05	−0.014	
	Hardness (N/m^2^)	10.09	> 0.05	0.04	
1	Adhesiveness (gf)	13.85	< 0.05	0.125	Fruits, Grains/Tubers, Mixed beans, Other, Vegetables
	Cohesiveness (ratio)	19.92	< 0.05	0.201	
	Hardness (N/m^2^)	17.05	< 0.05	0.165	
2	Adhesiveness (gf)	35.74	< 0.05	0.419	Eggs, Fruits, Grains/Tubers, Meats, Mixed beans, Other, Vegetables
	Cohesiveness (ratio)	38.23	< 0.05	0.454	
	Hardness (N/m^2^)	45.28	< 0.05	0.553	
3	Adhesiveness (gf)	38.99	< 0.05	0.446	Eggs, Fruits, Grains/Tubers, Meats, Mixed beans, Other, Vegetables
	Cohesiveness (ratio)	33.65	< 0.05	0.374	
	Hardness (N/m^2^)	35.77	< 0.05	0.402	
4	Adhesiveness (gf)	22.88	< 0.05	0.365	Eggs, Fruits, Grains/Tubers, Meats, Mixed beans, Vegetables
	Cohesiveness (ratio)	22.47	< 0.05	0.357	
	Hardness (N/m^2^)	24.74	< 0.05	0.403	
5	Adhesiveness (gf)	45.85	< 0.05	0.712	Eggs, Fruits, Grains/Tubers, Meats, Mixed beans, Other, Vegetables
	Cohesiveness (ratio)	25.44	< 0.05	0.347	
	Hardness (N/m^2^)	20.49	< 0.05	0.259	
6	Adhesiveness (gf)	34.06	< 0.05	0.395	Eggs, Fruits, Grains/Tubers, Meats, Mixed beans, Other, Vegetables
	Cohesiveness (ratio)	30.19	< 0.05	0.341	
	Hardness (N/m^2^)	17.86	< 0.05	0.167	
7	Adhesiveness (gf)	14.75	< 0.05	0.325	Eggs, Fruits, Grains/Tubers, Meats, Mixed beans, Vegetables
	Cohesiveness (ratio)	22.69	< 0.05	0.590	
	Hardness (N/m^2^)	27.82	< 0.05	0.761	

At Level 0, no significant differences were observed among food categories for any texture parameter (hardness, adhesiveness, or cohesiveness; all *p* > 0.05), with very small effect sizes (*ε* ^2^ ≤ 0.04). These findings indicate that liquid foods classified as Level 0 exhibited highly consistent mechanical properties regardless of food category, supporting a high degree of interchangeability at this level.

At Level 1, significant intra‐level differences emerged across all three texture parameters (*p* < 0.05), with small‐to‐moderate effect sizes (*ε* ^2^ = 0.125–0.201). Post hoc analyses revealed that grains and tubers exhibited higher hardness and cohesiveness than vegetables, whereas fruits showed lower adhesiveness compared with grains/tubers and vegetables (Table 5). These results indicate that even within early transitional textures, food category began to influence mechanical behavior.

**TABLE 5:** Dunn–Bonferroni significant pairwise comparisons within each IDDSI level (Bonferroni‐adjusted *p* and Cliff's *δ*).

Level	Metric	Cat i	Catj	n i	nj	*p* bonf	Cliffs delta
1	Hardness (N/m^2^)	Grains/Tubers	Vegetables	21	33	< 0.05	−0.691
1	Adhesiveness (gf)	Fruits	Grains/tubers	24	21	< 0.05	−0.333
1	Adhesiveness (gf)	Fruits	Vegetables	24	33	< 0.05	−0.265
1	Cohesiveness (ratio)	Fruits	Grains/tubers	24	21	< 0.05	−0.591
1	Cohesiveness (ratio)	Grains/Tubers	Vegetables	21	33	< 0.05	0.573
2	Hardness (N/m^2^)	Eggs	Meats	15	6	< 0.05	1
2	Hardness (N/m^2^)	Eggs	Grains/tubers	15	12	< 0.05	0.989
2	Hardness (N/m^2^)	Eggs	Mixed beans	15	6	< 0.05	1
2	Hardness (N/m^2^)	Fruits	Meats	15	6	< 0.05	1
2	Hardness (N/m^2^)	Meats	Vegetables	6	21	< 0.05	−0.968
2	Hardness (N/m^2^)	Eggs	Vegetables	15	21	< 0.05	0.867
2	Adhesiveness (gf)	Eggs	Grains/tubers	15	12	< 0.05	−0.9
2	Adhesiveness (gf)	Eggs	Meats	15	6	< 0.05	−1
2	Adhesiveness (gf)	Eggs	Mixed beans	15	6	< 0.05	−1
2	Adhesiveness (gf)	Eggs	Vegetables	15	21	< 0.05	−0.8
2	Cohesiveness (ratio)	Eggs	Meats	15	6	< 0.05	−1
2	Cohesiveness (ratio)	Eggs	Mixed beans	15	6	< 0.05	−1
2	Cohesiveness (ratio)	Eggs	Grains/tubers	15	12	< 0.05	−0.8
2	Cohesiveness (ratio)	Meats	Other	6	3	< 0.05	1
2	Cohesiveness (ratio)	Mixed beans	Other	6	3	< 0.05	1
3	Hardness (N/m^2^)	Grains/Tubers	Other	24	6	< 0.05	−1
3	Hardness (N/m^2^)	Meats	Other	6	6	< 0.05	−1
3	Hardness (N/m^2^)	Fruits	Grains/tubers	12	24	< 0.05	0.701
3	Hardness (N/m^2^)	Grains/Tubers	Vegetables	24	24	< 0.05	−0.573
3	Hardness (N/m^2^)	Fruits	Meats	12	6	< 0.05	1
3	Adhesiveness (gf)	Fruits	Grains/tubers	12	24	< 0.05	−0.875
3	Adhesiveness (gf)	Fruits	Meats	12	6	< 0.05	−1
3	Adhesiveness (gf)	Meats	Other	6	6	< 0.05	1
3	Adhesiveness (gf)	Grains/Tubers	Other	24	6	< 0.05	0.917
3	Adhesiveness (gf)	Fruits	Vegetables	12	24	< 0.05	−0.75
3	Cohesiveness (ratio)	Grains/Tubers	Other	24	6	< 0.05	0.972
3	Cohesiveness (ratio)	Meats	Other	6	6	< 0.05	1
4	Hardness (N/m^2^)	Eggs	Fruits	12	3	< 0.05	1
4	Hardness (N/m^2^)	Eggs	Meats	12	3	< 0.05	1
4	Adhesiveness (gf)	Grains/Tubers	Meats	13	3	< 0.05	−1
4	Adhesiveness (gf)	Fruits	Grains/tubers	3	13	< 0.05	1
4	Adhesiveness (gf)	Meats	Vegetables	3	21	< 0.05	1
4	Cohesiveness (ratio)	Meats	Mixed beans	3	3	< 0.05	1
4	Cohesiveness (ratio)	Fruits	Mixed beans	3	3	< 0.05	1
4	Cohesiveness (ratio)	Eggs	Meats	12	3	< 0.05	−1
5	Hardness (N/m^2^)	Eggs	Fruits	3	3	< 0.05	1
5	Hardness (N/m^2^)	Fruits	Mixed beans	3	6	< 0.05	−1
5	Adhesiveness (gf)	Meats	Mixed beans	12	6	< 0.05	1
5	Adhesiveness (gf)	Grains/Tubers	Meats	21	12	< 0.05	−0.944
5	Adhesiveness (gf)	Fruits	Mixed beans	3	6	< 0.05	1
5	Adhesiveness (gf)	Mixed beans	Other	6	6	< 0.05	−1
5	Adhesiveness (gf)	Fruits	Grains/tubers	3	21	< 0.05	1
5	Adhesiveness (gf)	Meats	Vegetables	12	12	< 0.05	1
5	Adhesiveness (gf)	Grains/Tubers	Other	21	6	< 0.05	−0.921
5	Cohesiveness (ratio)	Eggs	Fruits	3	3	< 0.05	−1
5	Cohesiveness (ratio)	Fruits	Other	3	6	< 0.05	1
5	Cohesiveness (ratio)	Fruits	Mixed beans	3	6	< 0.05	1
6	Hardness (N/m^2^)	Grains/Tubers	Other	21	6	< 0.05	−0.937
6	Hardness (N/m^2^)	Other	Vegetables	6	21	< 0.05	0.921
6	Adhesiveness (gf)	Grains/Tubers	Mixed beans	21	12	< 0.05	−0.865
6	Adhesiveness (gf)	Fruits	Grains/tubers	9	21	< 0.05	0.873
6	Adhesiveness (gf)	Eggs	Grains/tubers	6	21	< 0.05	0.81
6	Cohesiveness (ratio)	Mixed beans	Other	12	6	< 0.05	−1
6	Cohesiveness (ratio)	Grains/Tubers	Mixed beans	21	12	< 0.05	0.667
6	Cohesiveness (ratio)	Other	Vegetables	6	21	< 0.05	1
7	Hardness (N/m^2^)	Eggs	Fruits	3	12	< 0.05	−1
7	Hardness (N/m^2^)	Fruits	Vegetables	12	6	< 0.05	1
7	Hardness (N/m^2^)	Fruits	Meats	12	3	< 0.05	1
7	Adhesiveness (gf)	Fruits	Meats	12	3	< 0.05	−0.944
7	Cohesiveness (ratio)	Eggs	Mixed beans	3	3	< 0.05	1
7	Cohesiveness (ratio)	Fruits	Mixed beans	12	3	< 0.05	1
7	Cohesiveness (ratio)	Eggs	Vegetables	3	6	< 0.05	1
7	Cohesiveness (ratio)	Fruits	Vegetables	12	6	< 0.05	0.806

From Level 2 onward, intra‐level heterogeneity increased substantially. At Level 2, all texture parameters displayed highly significant differences among food categories, accompanied by large effect sizes (*ε* ^2^ = 0.419–0.553). Eggs emerged as a distinct category, exhibiting significantly higher hardness and significantly lower adhesiveness and cohesiveness compared with all other categories (Cliff's *δ* ranging from 0.867 to 1.000). Meats also differed significantly from vegetables in hardness, highlighting early divergence among solid–semi‐solid foods.

At Level 3, significant heterogeneity persisted across all parameters (*ε* ^2^ = 0.374–0.446). Differences in hardness were primarily associated with grains/tubers, which differed markedly from most other categories, whereas adhesiveness and cohesiveness differences reflected contrasting behaviors between meats, fruits, and grains/tubers. These patterns indicate increasing divergence in both resistance to deformation and internal structural integrity among foods sharing the same IDDSI classification.

At Level 4, intra‐level differences remained significant with moderate‐to‐large effect sizes (*ε* ^2^ = 0.357–0.403). Eggs consistently exhibited greater hardness than fruits and meats, whereas grains/tubers showed distinct adhesiveness patterns compared with meats and fruits. Differences in cohesiveness further distinguished eggs from meats and mixed beans, underscoring the persistence of category‐specific texture profiles at this level.

At Level 5, intra‐level variability reached its maximum magnitude, particularly for adhesiveness (*ε* ^2^ = 0.712). Multiple large pairwise differences were observed, including pronounced contrasts between mixed beans and other categories, as well as between grains/tubers and meats. In terms of cohesiveness, eggs exhibited significantly lower values than fruits, whereas fruits showed higher cohesiveness than mixed beans. These findings suggest that Level 5 represents a critical transition point at which food‐category‐specific texture differences become most pronounced.

At Levels 6 and 7, significant intra‐level differences across all three texture parameters persisted, with moderate‐to‐large effect sizes (*ε* ^2^ = 0.167–0.761). At Level 6, grains/tubers exhibited lower hardness than “other” foods but higher adhesiveness and cohesiveness compared with mixed beans. At Level 7, hardness differences were primarily driven by contrasts between eggs and fruits, as well as between fruits and vegetables, whereas cohesiveness differences further distinguished eggs and fruits from mixed beans and vegetables. These results indicate that even at the highest IDDSI levels, foods cannot be assumed to be mechanically interchangeable solely on the basis of categorical classification.

**FIGURE 7:**
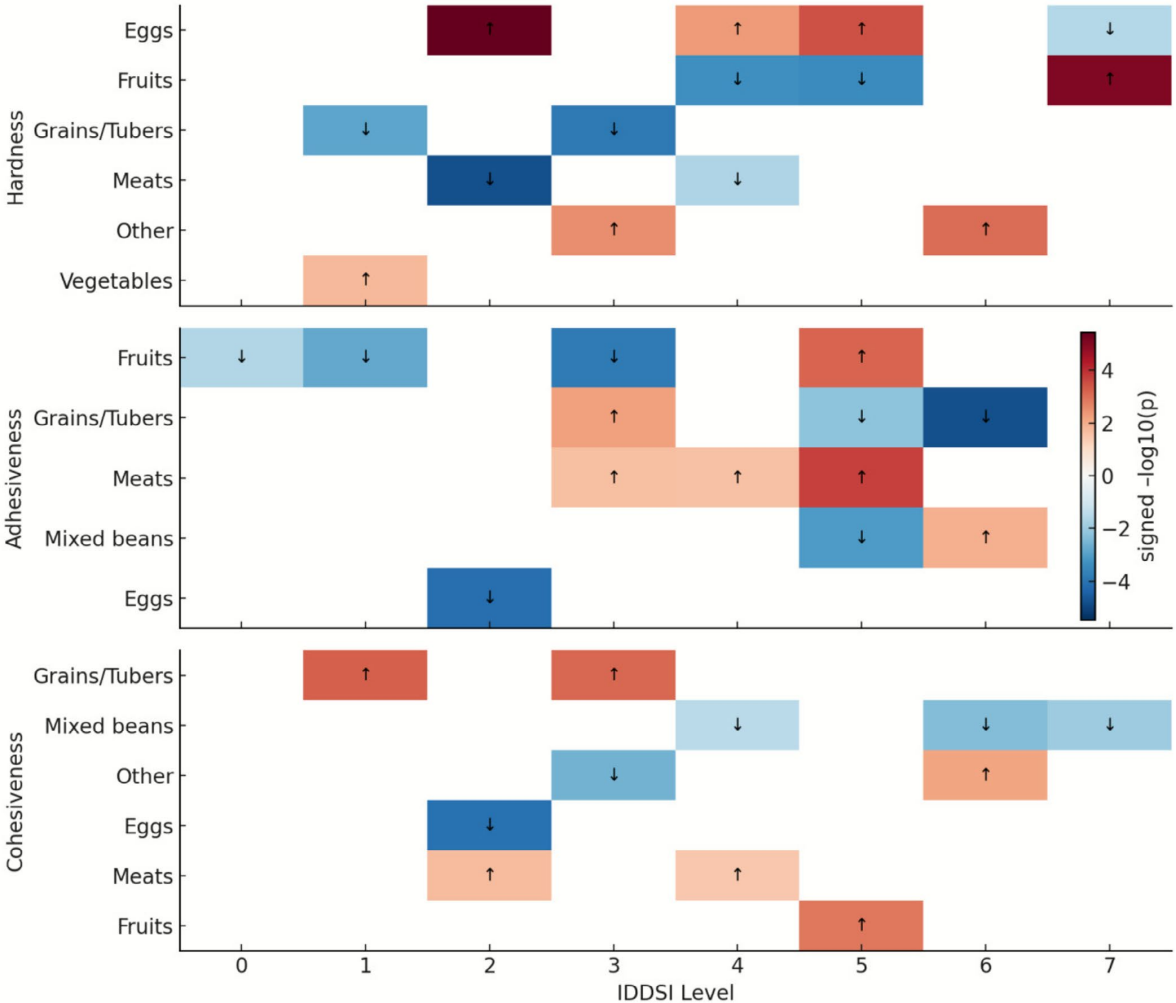
Categories deviating from level‐wide medians. (signed −log_10_ *p*; arrows show direction).

The direction and magnitude of category‐specific deviations from level‐wide medians are summarized in Figure 7, where signed −log_10_ (*p*) values indicate whether a given category exhibited higher or lower texture values relative to the median of the same IDDSI level. Eggs consistently showed positive deviations in hardness from Levels 2 to 5, whereas fruits and vegetables generally exhibited negative deviations. For adhesiveness, positive deviations were mainly concentrated in grains/tubers and meats, whereas fruits and eggs tended to show lower‐than‐median values. In terms of cohesiveness, grains/tubers and meats were frequently above the level‐wide median, whereas eggs and mixed beans were predominantly below it.

On the basis of the within‐level interchangeability assessment (Table 6), the number of recommended food‐category pairs decreased progressively with increasing IDDSI level, from 21 pairs at Level 0 to fewer than five pairs at higher levels. In parallel, the number of conditionally recommended and not recommended pairs increased markedly from Level 2 onward. For example, six food‐category pairs were classified as “not recommended” at Level 2, increasing to nine pairs at Level 5.

**TABLE 6:** Within‐level interchangeability of food‐category pairs by the IDDSI level (final decision with z‐distance).

Level	Recommended	Conditional	Not recommended
0	Eggs–Fruits; Eggs–Grains/Tubers; Eggs–Meats; Eggs–Mixed beans; Eggs–Other; Eggs–Vegetables; Fruits–Grains/Tubers; Fruits–Meats; Fruits–Mixed beans; Fruits–Other; Fruits–Vegetables; Grains/Tubers–Meats; Grains/Tubers–Mixed beans; Grains/Tubers–Other; Grains/Tubers–Vegetables; Meats–Mixed beans; Meats–Other; Meats–Vegetables; Mixed beans–Other; Mixed beans–Vegetables; Other–Vegetables	—	—
1	Fruits–Mixed beans; Fruits–Other; Grains/Tubers–Mixed beans; Mixed beans–Vegetables; Other–Vegetables	Fruits–Grains/Tubers; Fruits–Vegetables; Grains/Tubers–Other; Grains/Tubers–Vegetables; Mixed beans–Other	—
2	Eggs–Other; Fruits–Vegetables; Grains/Tubers–Meats; Grains/Tubers–Mixed beans; Grains/Tubers–Vegetables; Meats–Mixed beans	Eggs–Fruits; Fruits–Grains/Tubers; Fruits–Mixed beans; Fruits–Other; Grains/Tubers–Other; Mixed beans–Vegetables; Other–Vegetables	Eggs–Grains/Tubers; Eggs–Meats; Eggs–Mixed beans; Eggs–Vegetables; Fruits–Meats; Meats–Other; Meats–Vegetables; Mixed beans–Other
3	Eggs–Fruits; Eggs–Mixed beans; Eggs–Other; Eggs–Vegetables; Grains/Tubers–Meats; Mixed beans–Vegetables	Eggs–Grains/Tubers; Eggs–Meats; Fruits–Mixed beans; Fruits–Other; Grains/Tubers–Mixed beans; Meats–Mixed beans; Meats–Vegetables; Mixed beans–Other; Other–Vegetables	Fruits–Grains/Tubers; Fruits–Meats; Fruits–Vegetables; Grains/Tubers–Other; Grains/Tubers–Vegetables; Meats–Other
4	Eggs–Vegetables; Fruits–Meats; Grains/Tubers–Vegetables	Eggs–Grains/Tubers; Eggs–Mixed beans; Fruits–Vegetables; Grains/Tubers–Mixed beans; Mixed beans–Vegetables	Eggs–Fruits; Eggs–Meats; Fruits–Grains/Tubers; Fruits–Mixed beans; Grains/Tubers–Meats; Meats–Mixed beans; Meats–Vegetables
5	Grains/Tubers–Vegetables; Meats–Other	Eggs–Grains/Tubers; Eggs–Meats; Eggs–Mixed beans; Eggs–Other; Eggs–Vegetables; Fruits–Meats; Fruits–Vegetables; Grains/Tubers–Mixed beans; Mixed beans–Vegetables; Other–Vegetables	Eggs–Fruits; Fruits–Grains/Tubers; Fruits–Mixed beans; Fruits–Other; Grains/Tubers–Meats; Grains/Tubers–Other; Meats–Mixed beans; Meats–Vegetables; Mixed beans–Other
6	Fruits–Mixed beans; Fruits–Vegetables; Meats–Vegetables; Mixed beans–Vegetables	Eggs–Fruits; Eggs–Meats; Eggs–Mixed beans; Eggs–Other; Eggs–Vegetables; Fruits–Meats; Fruits–Other; Grains/Tubers–Meats; Grains/Tubers–Vegetables; Meats–Mixed beans; Meats–Other	Eggs–Grains/Tubers; Fruits–Grains/Tubers; Grains/Tubers–Mixed beans; Grains/Tubers–Other; Mixed beans–Other; Other–Vegetables
7	—	Eggs–Grains/Tubers; Eggs–Meats; Fruits–Grains/Tubers; Grains/Tubers–Meats; Grains/Tubers–Mixed beans; Grains/Tubers–Vegetables; Meats–Mixed beans; Meats–Vegetables; Mixed beans–Vegetables	Eggs–Fruits; Eggs–Mixed beans; Eggs–Vegetables; Fruits–Meats; Fruits–Mixed beans; Fruits–Vegetables

Collectively, these results demonstrate that although foods at Level 0 are largely interchangeable, significant category‐dependent texture differences emerge from Level 1 onward and become most pronounced at intermediate IDDSI levels (Levels 2–5). This pronounced intra‐level heterogeneity highlights the limitations of relying solely on IDDSI classification for food substitution and underscores the necessity of food‐category‐specific texture evaluation when designing dysphagia‐friendly diets.

## 4 Discussion

### 4.1 Variations in Textural Characteristics Across IDDSI Levels

This study quantitatively demonstrated systematic variations in hardness (H), adhesiveness (A), and cohesiveness (C) across IDDSI Levels 0–7 in foods commonly consumed by elderly individuals. Non‐parametric analyses confirmed that all three texture parameters differed significantly across levels, with large effect sizes for hardness (*η* ^2^ = 0.81), adhesiveness (*η* ^2^ = 0.54), and cohesiveness (*η* ^2^ = 0.67). In addition, monotonic trends identified by the Jonckheere–Terpstra test further supported the hierarchical organization of the IDDSI framework, indicating that these texture parameters change in an ordered manner along the liquid–solid continuum.

From a functional perspective, hardness showed a clear progressive increase from Level 0 to Level 7, reflecting the transition from flowable liquids to textures requiring cutting or mastication. In contrast, adhesiveness followed a non‐linear trajectory, with the most negative values concentrated at intermediate levels (Levels 3–5) before decreasing in absolute magnitude at higher levels. This pattern suggests that semi‐solid foods reach peak surface stickiness during transitional gelation and water‐binding stages, whereas firmer textures rely more on structural rigidity than surface adhesion (Xie et al. 2018; Su et al. 2018; Giura et al. 2021). Cohesiveness declined gradually with increasing IDDSI level, indicating progressive weakening of internal bonding strength and an increased tendency for structural breakdown under compression (Godschalk‐Broers et al. 2022; Fu et al. 2023).

Taken together, these results confirm that the IDDSI framework effectively captures the progressive transformation of food texture from liquid to solid states. Importantly, the observed increase in hardness and concomitant decrease in cohesiveness were obtained using naturally prepared foods without commercial thickeners or gelling agents. This provides objective validation that the IDDSI hierarchy reflects intrinsic structural changes arising from common domestic food preparation methods, rather than being limited to industrially formulated products (Tong et al. 2023; Muxi et al. 2024; Chen, Cheng, et al. 2025; Chen, Mu, et al. 2025).

### 4.2 Intra‐Level Variability and Non‐Interchangeability Among Food Categories

Although foods from different categories may satisfy the same IDDSI classification criteria, their mechanical properties were far from uniform. Significant intra‐level differences were detected for at least one texture parameter at every IDDSI level, with the greatest heterogeneity observed at intermediate levels (Levels 3–5). These findings directly support the premise that classification within the same IDDSI level does not imply functional interchangeability (Chen, Cheng, et al. 2025; Chen, Mu, et al. 2025).

At lower levels (Levels 1–2), category‐dependent differences began to emerge, particularly between grains/tubers and vegetables. By Level 2, eggs displayed pronounced divergence from other food categories across all three texture parameters, whereas meats and vegetables also showed substantial separation in hardness. As foods progressed to Levels 3–4, inter‐category divergence became more pronounced and structured: grains/tubers and meats exhibited near‐complete separation in hardness, fruits and vegetables differed markedly in adhesiveness, and meats and mixed beans showed large differences in cohesiveness. At higher levels (Levels 5–6), effect sizes approached their theoretical maximum, indicating almost complete mechanical separation between certain food categories (Giura et al. 2021; Wang et al. 2019).

This progressive divergence was further reflected in the interchangeability assessment. Although all category pairs at Level 0 were classified as “recommended,” indicating high uniformity, the number of “conditional” and “not recommended” pairs increased sharply from Level 1 onward. This trend was especially evident between protein‐rich and fiber‐rich foods, such as meats versus vegetables or eggs versus mixed beans. By Level 5, nearly half of the possible category pairs were deemed ‘not recommended,’ and at Level 7, most substitutions required conditional adjustment (Chen, Cheng, et al. 2025; Hernández et al. 2025).

Visualization of signed –log_10_(*p*) deviations further revealed consistent category‐specific clustering patterns. Eggs and meats tended to exhibit positive deviations in hardness, whereas fruits and vegetables were characterized by lower adhesiveness values. In terms of cohesiveness, grains/tubers and meats generally remained above the level‐wide median, whereas mixed beans and eggs were below. Collectively, these results indicate that intra‐level variability is not random but reflects systematic differences in food composition and structure (Hadde et al. 2022; Su et al. 2018; Giura et al. 2021). Consequently, although foods may meet identical IDDSI flow or compression criteria, their oral processing behaviors remain inherently distinct, underscoring the limitation of relying on IDDSI level alone to infer functional equivalence (Muxi et al. 2024; Qin et al. 2025).

### 4.3 Mechanistic Interpretation of Textural Differences

The observed inter‐category textural variations can be attributed to compositional and physicochemical mechanisms. Starch‐based foods (e.g., rice, sweet potato, potato) are primarily influenced by gelatinization and retrogradation. Gelatinization increases viscosity and softness, whereas retrogradation induces rigidity and brittleness, explaining the higher adhesiveness of mashed potatoes (Level 5) and the increased hardness but reduced stickiness of steamed rice (Level 7) (Wang et al. 2019; Qin et al. 2025; Hernández et al. 2025).

Protein‐based foods (e.g., pork, beef, fish, and eggs) undergo denaturation and crosslinking during heating, forming resilient gel networks with high elasticity and cohesiveness. These structures enhance bolus formation while reducing surface adhesiveness, facilitating smoother swallowing (Su et al. 2018; Giura et al. 2021; Chen, Mu, et al. 2025; Lu et al. 2025).

In contrast, fiber‐rich vegetables and legumes (e.g., celery, carrot, and red bean) contain substantial cellulose and hemicellulose, resulting in incomplete cell wall breakdown during blending. The residual fibrous microstructure leads to high adhesiveness and low cohesiveness, increasing the risk of oral residue and post‐swallow retention (Dong et al. 2023; Xie et al. 2018; Tong et al. 2023; Qin et al. 2025). Water content also plays a dual role in texture modulation. Moderate hydration promotes balance between hardness and adhesiveness, whereas excessive or insufficient moisture disrupts matrix stability, influencing flow behavior and swallowing safety (Godschalk‐Broers et al. 2022; Fu et al. 2023; Chen, Cheng, et al. 2025).

### 4.4 Practical Implications for Dysphagia Diet Design

These findings have direct implications for the rational design of texture‐modified diets for dysphagia management. First, the consistent increase in hardness and decrease in cohesiveness across IDDSI levels provide quantifiable indicators for evaluating IDDSI compliance in naturally prepared foods (Cichero et al. 2017; Wong et al. 2023; Hadde et al. 2022). Second, the pronounced intra‐level heterogeneity highlights the need for ingredient‐specific texture matching rather than simple level‐based substitution.

According to the interchangeability criteria proposed in this study, foods with non‐significant differences across all three parameters (H/A/C) and a Euclidean distance ≤ 0.6 can be considered “recommended interchangeable.” Examples include fruit–vegetable pairs at Levels 0–2 (e.g., apple purée and pumpkin purée) and meat–grain/tuber pairs at Levels 6–7 (e.g., minced chicken and soft steamed rice) (Muxi et al. 2024; Chen, Cheng, et al. 2025; Chen, Mu, et al. 2025). In contrast, high‐adhesiveness and low‐cohesiveness foods, such as mixed beans or fibrous vegetables at Levels 3–5, should not be used as substitutes for protein‐ or starch‐based items, as they may increase oral residue and aspiration risk (Giura et al. 2021; Tong et al. 2023; Qin et al. 2025).

Importantly, the present results demonstrate that traditional Chinese cooking methods—such as steaming, boiling, and blending with controlled water ratios—can achieve IDDSI‐compliant textures without reliance on synthetic thickeners (Dong et al. 2023; Muxi et al. 2024; Hernández et al. 2025). This finding supports the feasibility of culturally appropriate, cost‐effective strategies for long‐term care facilities and home‐based dysphagia nutrition management among older adults (Chen, Cheng, et al. 2025; Chen, Mu, et al. 2025; Lu et al. 2025).

### 4.5 Overall Significance

This study provides the first comprehensive quantitative evidence describing the texture evolution of commonly consumed Chinese foods across the full IDDSI 0–7 continuum. The findings reveal a coexistence of cross‐level regularity and intra‐level heterogeneity:

1. The IDDSI framework effectively captures progressive textural transitions in hardness, adhesiveness, and cohesiveness;
2. Foods classified under the same level may differ substantially in structural and swallowing behavior, challenging the notion of equivalence;
3. Incorporating three‐dimensional texture parameter analysis (H/A/C) enables objective interchangeability assessment, bridging the gap between laboratory measurement, domestic preparation, and clinical practice.

By establishing an empirical foundation for texture–function matching in natural food matrices, this research contributes to the scientific localization of the IDDSI system and supports the development of safe, culturally compatible dysphagia diets tailored to the Chinese elderly population.

## 5 Conclusion

This study systematically characterized the texture profiles of foods commonly consumed by elderly individuals in China within the IDDSI framework and established quantitative relationships among hardness, adhesiveness, and cohesiveness across Levels 0–7. The results demonstrate that hardness increases progressively with higher IDDSI levels, cohesiveness decreases accordingly, and adhesiveness follows a non‐linear pattern with a pronounced peak in the semi‐solid range (Levels 3–5). These consistent trends confirm that naturally prepared foods, even in the absence of commercial thickeners or gelling agents, can reproducibly exhibit measurable textural gradients aligned with IDDSI classifications.

Beyond cross‐level trends, substantial within‐level heterogeneity was observed among food categories, indicating that foods assigned to the same IDDSI level are not functionally interchangeable. In particular, fiber‐rich vegetables and legumes exhibited higher adhesiveness and lower cohesiveness than protein‐based foods, suggesting a greater potential for oral residue and compromised bolus control. These findings highlight that although IDDSI levels effectively describe macroscopic texture transitions, they do not fully capture ingredient‐specific microstructural and compositional factors that influence swallowing performance.

By integrating non‐parametric statistical analyses with effect size estimation, this study further proposes a quantitative framework for within‐level substitution, using centroid distance (≤ 0.60) as an operational criterion for assessing interchangeability. This approach provides practical guidance for clinicians, caregivers, and food preparers in selecting culturally appropriate, dysphagia‐safe food alternatives while maintaining IDDSI compliance.

Overall, this work bridges the gap between the international IDDSI framework and natural Chinese food systems. By providing empirical evidence for the applicability of IDDSI standards to homemade foods, the study contributes to the localization and refinement of dysphagia diet design. Future research should incorporate microstructural characterization, rheological modeling, and clinical swallowing assessments to develop predictive models linking food composition, texture, and swallowing safety.

## Funding

This work was supported by the Department of Science and Technology of Guangdong Province, China (Grant No. JH2019045), and by the National Key Research and Development Program of China (Grant No. 2023YFF1104405).

## Supporting information


**Tables S1–S2:** fsn371467‐sup‐0001‐TablesS1‐S2.docx.

## Data Availability

The data that support the findings of this study are available from the corresponding author upon reasonable request.
