# Supplementary material for: Comparative Study on the Hardness, Adhesiveness, and Cohesiveness of Ingredients on the Basis of IDDSI Levels and Ingredient Selection
Source: Food Sci Nutr. 2026 Jan 19;14(1):e71467. doi: 10.1002/fsn3.71467 (PMC12813622; doi:10.1002/fsn3.71467)
Supplement: Supplementary file 1 — Tables S1–S2: fsn371467‐sup‐0001‐TablesS1‐S2.docx. [file FSN3-14-e71467-s001.docx]

**Supplementary Table S1. Selected ingredients and food categories**

| **Food category** | **Ingredient** |
| --- | --- |
| Meats | Pork, Beef, Chicken, Fish |
| Eggs | Hen’s egg |
| Grains & tubers | Rice, Noodles, Oats, Potato, Sweet potato, Yam, Pumpkin |
| Vegetables | Tomato, Eggplant, Cabbage, Carrot, Chinese greens, Broccoli, Spinach |
| Mixed beans | Red bean, Mung bean |
| Fruits | Apple, Pear, Peach, Banana |
| Others | Tofu |

**Supplementary Table S2. Ingredient-specific processing parameters**

| **Food category** | **Ingredient** | **Pre-treatment** | **Cooking method** | **Cooking time** | **Water ratio**  **(w/w)** |
| --- | --- | --- | --- | --- | --- |
| Meat | Pork | Trim visible fat and connective tissue; cut into 2 × 2 cm cubes | Steam | 30 min | 1:0, 1:1, 1:2, 1:3 |
|  | Chicken | Trim fat and tendons; cut into 2 × 2 cm cubes | Steam | 30 min | 1:1, 1:2, 1:3 |
|  | Beef | Trim fat and sinew; cut into 2 × 2 cm cubes | Steam | 30 min | 1:0, 1:1, 1:2 |
|  | Fish | Debone and remove skin; cut into 2 × 2 cm cubes | Steam | 30 min | 1:1, 1:2, 1:3 |
| Eggs | Egg yolk (boiled) | Boil whole egg, separate yolk | Boil | 20 min | 1:0.5, 1:1, 1:1.5, 1:2 |
|  | Steamed egg | Beat whole egg with water | Steam | 20 min | 1:1.5, 1:2, 1:2.5, 1:3 |
| Grains | Rice | Soak 30 min before cooking | Steam | 30 min | 1:4, 1:3, 1:2, 1:1 |
|  | Oats | – | Boil | 20 min | 1:4, 1:6, 1:8, 1:10, 1:12 |
|  | Wheat paste | Mix flour with water | Steam | 20 min | 1:0.5, 1:1, 1:1.5, 1:2 |
|  | Noodles | Boil, drain, mince | Boil | 8 min | 1:0.5, 1:1 |
| Tubers | Sweet potato | Peel; cut into cubes | Steam | 30 min | 1:0, 1:0.5, 1:1, 1:1.5, 1:2, 1:2.5 |
|  | Potato | Peel; cut into cubes | Steam | 30 min | 1:0, 1:0.5, 1:1, 1:1.5, 1:2, 1:2.5 |
|  | Yam | Peel; cut into cubes | Steam | 30 min | 1:0, 1:0.5, 1:1, 1:1.5, 1:2, 1:2.5 |
| Vegetables | Carrot | Wash; dice | Steam | 30 min | 1:0.5, 1:1, 1:1.5, 1:2, 1:2.5 |
|  | Broccoli | Remove stem; dice | Steam | 30 min | 1:0.5, 1:1, 1:1.5, 1:2, 1:2.5 |
|  | Chinese cabbage | Remove outer leaves; dice | Steam | 30 min | 1:0, 1:0.5, 1:1, 1:1.5, 1:2, 1:2.5 |
|  | Spinach | Remove roots; chop | Steam | 10 min | 1:0.5, 1:1, 1:1.5, 1:2, 1:2.5 |
|  | Tomato | Wash; dice | Steam | 10 min | 1:0, 1:0.5, 1:1, 1:1.5, 1:2 |
|  | Eggplant | Peel; dice | Steam | 30 min | 1:0.5, 1:1, 1:1.5, 1:2, 1:2.5 |
|  | Pumpkin | Peel; dice | Steam | 30 min | 1:0, 1:0.5, 1:1, 1:1.5, 1:2, 1:2.5 |
| Mixed beans | Red bean | Soak 12 h | Steam | 30 min | 1:0, 1:0.5, 1:1, 1:1.5, 1:2, 1:2.5 |
|  | Mung bean | Soak 12 h | Steam | 30 min | 1:0, 1:0.5, 1:1, 1:1.5, 1:2, 1:2.5 |
| Fruits | Apple | Peel and core | Steam / blend | 10 min | 1:0, 1:0.5, 1:1, 1:1.5 |
|  | Pear | Peel and core | Steam / blend | 10 min | 1:0, 1:0.5, 1:1, 1:1.5 |
|  | Peach | Peel and pit | Blend (no heat) | 0 min | 1:0.5, 1:1, 1:1.5 |
|  | Banana | Peel | Blend (no heat) | 0 min | 1:0, 1:0.5, 1:1, 1:1.5, 1:2 |
| Soy products | Tofu | Drain excess water | Steam | 10 min | 1:0, 1:0.5, 1:1, 1:1.5, 1:2 |

**Table footnote**

Cooking time refers to the duration of thermal processing prior to blending. For raw-blended fruits, cooking time is defined as 0 min. Water ratio (w/w) indicates the mass ratio of food material to added water; a ratio of 1:0 denotes no additional water.
